# *Lip*, a Human Gene Detected by Transfection of DNA From a Human Liposarcoma Encodes a Protein With Homology to Regulators of Small G Proteins

**DOI:** 10.1080/13577149878145

**Published:** 1998-03

**Authors:** Helen Patterson, Sandra Gill, Harry Benjamin, Philip J. Mitchell, Colin S. Cooper

**Affiliations:** 1 Section of Molecular Carcinogenesis Institute of Cancer Research 15 Cotswold Road Sutton, Surrey Belmont SM2 5NG UK; 2 Cell Biology and Experimental Pathology Institute of Cancer Research Surrey UK

## Abstract

*Purpose/Method.* Transfection experiments have been used to identify activated oncogenes in a wide variety of tumour types. Here we describe the use of transfection experiments utilizing DNA from a human pleomorphic liposarcoma to identify a novel gene, designated *lip* which maps to chromosome 19.

*Results. lip* was expressed in all sarcoma cell lines examined and a wide variety of normal tissues. Sequencing of cDNAs prepared from transcripts of the normal *lip* gene indicates that *lip* is predicted to encode a 966 amino acid protein with a region of homology to proteins such as *vav, dbl, lbc* and *ect-2* which act as GDP–GTP exchange factors for the RAS superfamily of small GTP-binding proteins, and the N-terminal 830 amino acids are identical to the recently identified gene p115-RhoGEF, an exchange factor for RHOA. In transfectants, *lip* has undergone a rearrangement which results in C-terminal truncation of the predicted LIP protein. However, we failed to detect this alteration in the primary liposarcoma used in the original transfection experiments, or in other sarcoma specimens examined.

*Discussion.* When considered together, these observations suggest that transforming *lip* sequences represent an alternatively spliced form of p115-RhoGEF that is activated for transformation by C-terminal truncation during transfection, and is not widely involved in sarcoma development.


*Sarcoma* (1998) **2**, 35–44

ORIGINAL ARTICLE

### *lip*, a human gene detected by transfection of DNA from a human liposarcoma encodes a protein with homology to regulators of small G proteins

HELEN PATTERSON,[1,2] SANDRA GILL,[1] HARRY BENJAMIN,[1] PHILIP J. MITCHELL[2] & COLIN S. COOPER[1]

*Section of [1]Molecular Carcinogenesis and [2]Cell Biology and Experimental Pathology, Institute of Cancer Research, Surrey, UK*

**Abstract**
*Purpose/Method.* Transfection experiments have been used to identify activated oncogenes in a wide variety of tumour types. Here we describe the use of transfection experiments utilizing DNA from a human pleomorphic liposarcoma to identify a novel gene, designated *lip* which maps to chromosome 19.
*Results.* *lip* was expressed in all sarcoma cell lines examined and a wide variety of normal tissues. Sequencing of cDNAs prepared from transcripts of the normal *lip* gene indicates that *lip* is predicted to encode a 966 amino acid protein with a region of homology to proteins such as *vav*, *dbl*, *lbc* and *ect-2* which act as GDP–GTP exchange factors for the RAS superfamily of small GTP-binding proteins, and the N-terminal 830 amino acids are identical to the recently identified gene p115-RhoGEF, an exchange factor for RHOA. In transfectants, *lip* has undergone a rearrangement which results in C-terminal truncation of the predicted LIP protein. However, we failed to detect this alteration in the primary liposarcoma used in the original transfection experiments, or in other sarcoma specimens examined.
*Discussion.* When considered together, these observations suggest that transforming *lip* sequences represent an alternatively spliced form of pll5-RhoGEF that is activated for transformation by C-terminal truncation during transfection, and is not widely involved in sarcoma development.

**Key words**: *transfection, soft tissue sarcoma, nucleotide exchange factor.*

### Introduction

Transfection of DNA into NIH3T3 mouse fibroblasts has been used to survey a wide variety of human tumours for the presence of transforming oncogenes. Although the majority of the genes detected by this assay are activated *ras* genes, a number of other genes including *met*,[1] *ret*, *trk*,[3] *mas*[4], *dbl*,[5] *raf*,[6] *hst*,[7] *vav*,[8] *ufo/axl*,[9,10] *ect–2*[11] and *lbc*[12] have also been identified. These genes encode proteins of several functional classes, including growth factors, transmembrane receptors with tyrosine kinase activity. non-receptor serine–threonine kinases and regulators of small GTP-binding proteins, all of which are thought to play a role in intracellular signalling pathways which regulate cellular proliferation.

In an attempt to identify oncogenes activated in human soft tissue tumours, DNA from 29 sarcomas was examined for the ability to transform NIH3T3 cells.[13] These studies identified an activated *k-ras* gene in a leiomyosarcoma, and a novel activated gene following transfection of DNA from a pleomorphic liposarcoma. Genomic fragments of this novel gene, designated *lip*, were cloned by screening a genomic library prepared using DNA from a *lip* secondary transfectant with a human *alu*-repeat probe. Repeat-free subclones of these genomic clones have been used to demonstrate that this gene maps to chromosome 19 and is expressed as a 3.0-kb transcript in primary and secondary *lip* transfectants.[13]

cDNAs corresponding to the normal *lip* gene have now been cloned from a normal fibroblast cDNA library and through sequencing analysis were found to encode a protein with regions of homology to proteins such as exchange factors for small GTP-binding proteins. In addition the N-terminal 830 amino acids are almost completely identical to the recently cloned p115-RhoGEF.[14] Sequencing of cDNA clones isolated from a primary transfectant

Correspondence to: H. Patterson, Section of Molecular Carcinogenesis, Institute of Cancer Research, 15 Cotswold Road, Belmont, Sutton, Surrey, SM2 5NG, UK. Tel: +44 181 643 8901; Fax: +44 181 770 7290.

1357-714X/98/040035-10 ©1998 Carfax Publishing Ltd library indicate that 3′ sequences are lost during its activation.

### Materials and Methods

### *5′ RACE polymerase chain reaction analysis*

Cytoplasmic RNA was extracted from subconfluent cultures of MCF-7 cells.[15] 5′ RACE (rapid amplification of cDNA ends) was performed as recommended using the 5′ RACE System (Gibco BRL), with the exception that RNA was reverse transcribed using random hexamer primers (Pharmacia). Polymerase chain reaction (PCR) primers were as follows: first round of amplification GAGTTT-GTCTCCAGCTCG, second round of amplification CTCAAAATCCTCATCCTCAGC.

### *Preparation and screening of cDNA libraries*

cDNA clones corresponding to the normal *LIP* gene were obtained by screening a randomly primed HT1080 cDNA library[16] and an oligo-dT-primed M426 human fibroblast cDNA library[17] Clones corresponding to the transfected gene were obtained from a primary transfectant cDNA library constructed as follows. RNA was extracted from subconfluent cultures of transfectant cells,[18] and poly(A)$^+$ RNA selected using oligo-(dT)-cellulose (Poly(A) Quik Kit, Stratagene). The cDNA library was constructed in the lambda ZAP II vector (Stratagene) using the ZAP II cDNA synthesis kit (Stratagene). All three libraries were screened using Biodyne hybridization membranes (Pall-Biodyne) as previously described.[16]

### *cDNA sequencing*

Partially deleted subclones of the cDNA inserts were generated using exonuclease III and mung bean nuclease (Stratagene), and these subclones sequenced by the dideoxy chain termination method[19] using the Sequenase Version 2.0 sequencing kit (United States Biochemicals).

Alternatively, primary transfectant poly(A)$^+$ RNA was reverse transcribed using random hexamer primers (Pharmacia) and Superscript (BRL), and primers derived from the normal *lip* sequence were used to amplify 20 ng of cDNA in 25 $\mu$l of *pfu* buffer (Stratagene) containing 10 pmoles each primer, 200 $\mu$M each dNTP and 0.5 units of *pfu* DNA polymerase (Stratagene), with 30 cycles of 1 min at 92$^\circ$, 1 min at 55$^\circ$ and 3 min at 72$^\circ$. PCR products, purified by gel electrophoresis and Geneclean (Bio101 Inc.), were digested with XhoI and XbaI before being cloned into XhoI/XbaI cut Bluescript (SK+). Inserts sequenced as already described. Primers pairs were as follows:

Primer 1: TACGCTCGAGACTTCTACCACAGCTTCCTG
and
Primer 2: CGTACTCGAGACATCTTCCCCAGCCTGGAC
Primer 3: GTCATCTAGAGCTATGTGACTGTACTCCAG
and
Primer 4A: CATGTCTAGAACTCCCTGAACCTCCAGCTC.

### Results

### *Isolation and sequencing of cDNA clones*

The genomic clone MC15, isolated in our previous study,[13] was used to screen an oligo-dT primed M426 human fibroblast library,[17] and two clones of 3.0 kb (11A) and 2.6 kb (13A) were isolated. Complete bidirectional sequencing of clone 11A generated a sequence of 3023 bp including a 39-bp polyA tail. Although this clone corresponded in size to the 3.0 kb transcript detected by Northern analysis in a variety of human tumour cell lines (data not shown), the open reading frame present in this cDNA extended to the 5′ end of the sequence, raising the possibility that an additional 5′ sequence existed. The same probe was therefore used to screen 100 000 clones from a randomly primed HT1080 cDNA library and three additional clones 12C, 5A and 3B were isolated. Of these, 3B contained a 2.6-kb insert, was primed at nucleotide 2542 with respect to 11A and possessed an alternative 5′ end (Fig. 1(a)). Subsequent 5′ RACE analysis, using RNA derived from a human breast cell line, was used to demonstrate that 18 bp of the 5′ sequence was missing from the original clone 11A. This upstream sequence, GAGGCTTCGGTTCCG-GTG, did not, however, encode an upstream stop codon or an initiating methionine. In clone 11A, there are two methionines at the 5′ terminus which conform closely to the Kozak consensus sequence initiating at methionine,[20] the first 7 bp and the second 52 bp from the 5′ end of this clone. Neither is preceded by an in-frame stop codon. In clone 3B the methionine at position 7 is absent (Fig. 2(a)). We have therefore chosen the methionine at position 52 as the most likely translation start site since this is present in both 11A and 3B. If this is the case, *lip* encodes a single major open-reading frame of 2898 nucleotides predicting a protein of 966 amino acids. The sequence and the putative open-reading frame are shown in Fig. 2(b).

### *lip expression*

*lip* was expressed as a 3-kb transcript in all cell lines examined. These included the fibrosarcoma cell lines HT1080 and Hs913T, the leiomyosarcoma lines SK-UT-1 and SK-LMS-1, the rhabdomyosarcoma cell lines A204, RMS and RD, the Ewings sarcoma cell line A673, the promyelocytic leukaemia cell line HL-60 and the carcinoma cell line A431. In addition, *lip* was shown to be expressed in a variety of tissues including tonsil, spleen, renal cortex, lung, prostate, endometrium and breast (data not shown).

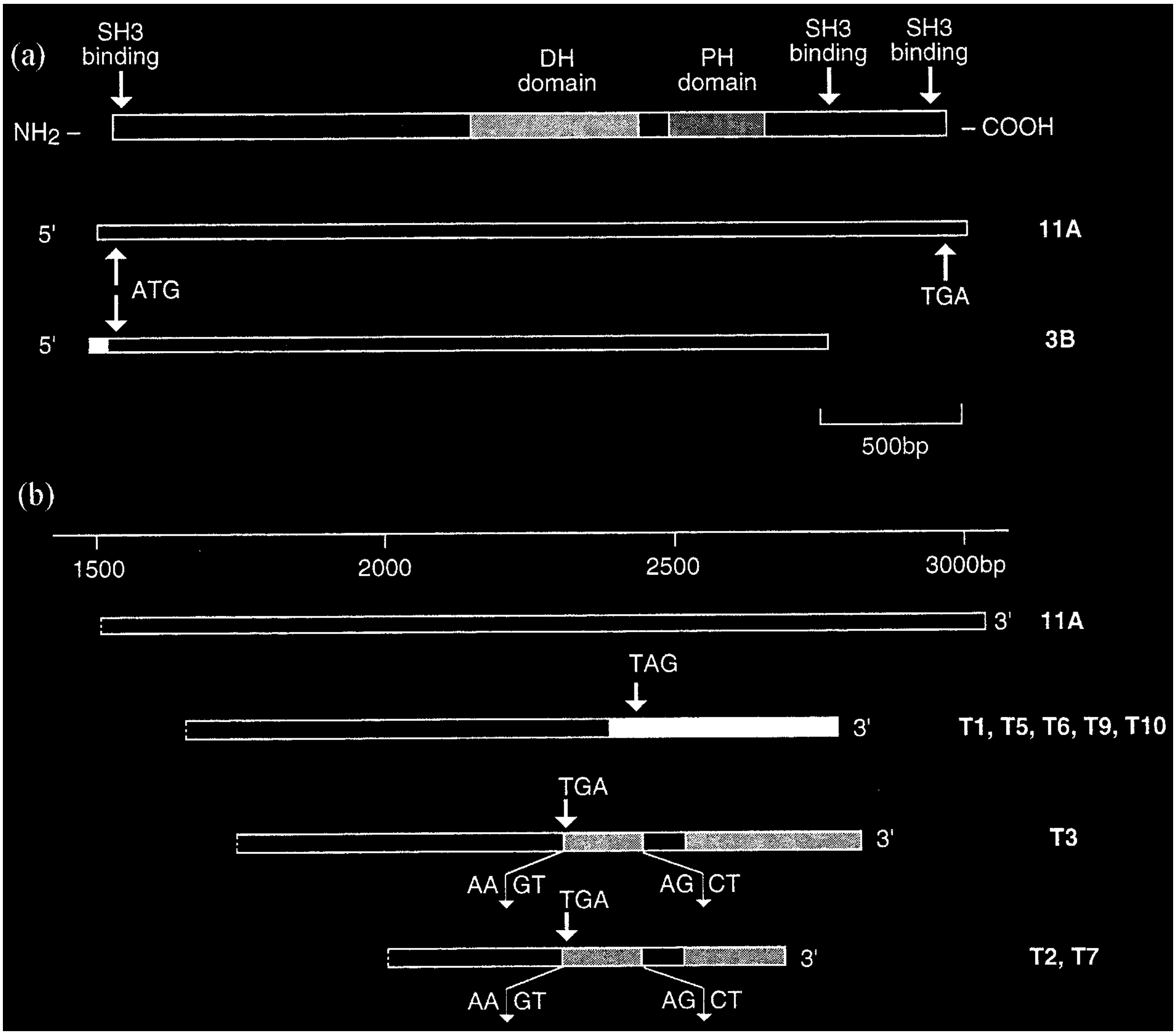

**Fig. 1.** *(a) Schematic representation of the predicted lip protein, the cDNA clones 11A and 3B. The Kozak methionine codon, ATG, present in both clones is marked, as is the in and frame stop codon. The alternative 5′ sequence in clone 3B is demonstrated. The dbl homology (DH) and pleckstrin homology (PH) domains and the potential SH3 domain-binding sites are also shown. (b) Schematic representation of the 3′ ends of clone 11A, transfectant clones T1, T5, T6, T9, T10, and T3, T2 and T7. Clones are aligned with respect to clone 11A. The alternative 3′ sequences in each of the three groups of transfectant clones are demonstrated by shading. The sequence at the junction sites between lip, non-lip sequences in transfectant clones T2, T3, T7 is shown and indicates that the 138-bp sequence inserted between lip nucleotides 2300 and 2301 may be a retained intron.*

*The predicted lip protein contains nucleotide exchange factor and PH domains*

The predicted *lip* protein contains regions of moderate similarity to the previously described *dbl* homology (DH) domain seen in the transforming oncogenes *dbl*, *vav*, *ect-2* and *tim*;[21] the yeast cell cycle gene *cdc24*, the *bcr* gene[22] and a nucleotide exchange factor for *ras*, p140-RasGRF.[23] Using the FASTA search programme, the core region of this DH domain (amino acids 416–610) was most closely related to the *lbc*, *vav*, *ect-2* and *cdc24* genes, demonstrating 33.8%, 25.9%, 25.5% and 24.3% identity respectively (Fig. 3(a)).

A second region of similarity to the pleckstrin homology (PH) domain[24] has been found to span amino acids 649 to 758. This domain, first identified as an internal repeat in pleckstrin, the major substrate of protein kinase C in platelets,[25] has been identified in a number of other proteins including the products of the *vav*, *dbl*, *rasgrf*, *bcr*, *lbc* and *cdc24* genes. *lip* shows 24% identity with the pleckstrin C-terminal PH domain. Although the identity is low, the family members noted so far have exhibited only 21–25% identity. All five of the previously defined subdomains can be identified, and the most highly conserved residues which define these subdomains[25] are also conserved in *lip* (Fig. 3(b)).

The C-terminal 200 amino acids are relatively proline rich (16%) and contain a number of sites which conform to the minimal consensus sequence for SH3 domain binding sites, P–*/p–X–P (P = proline, */p = usually hydrophobic/proline, X = not conserved).[26] There is an additional potential SH3 domain-binding sequence in the amino

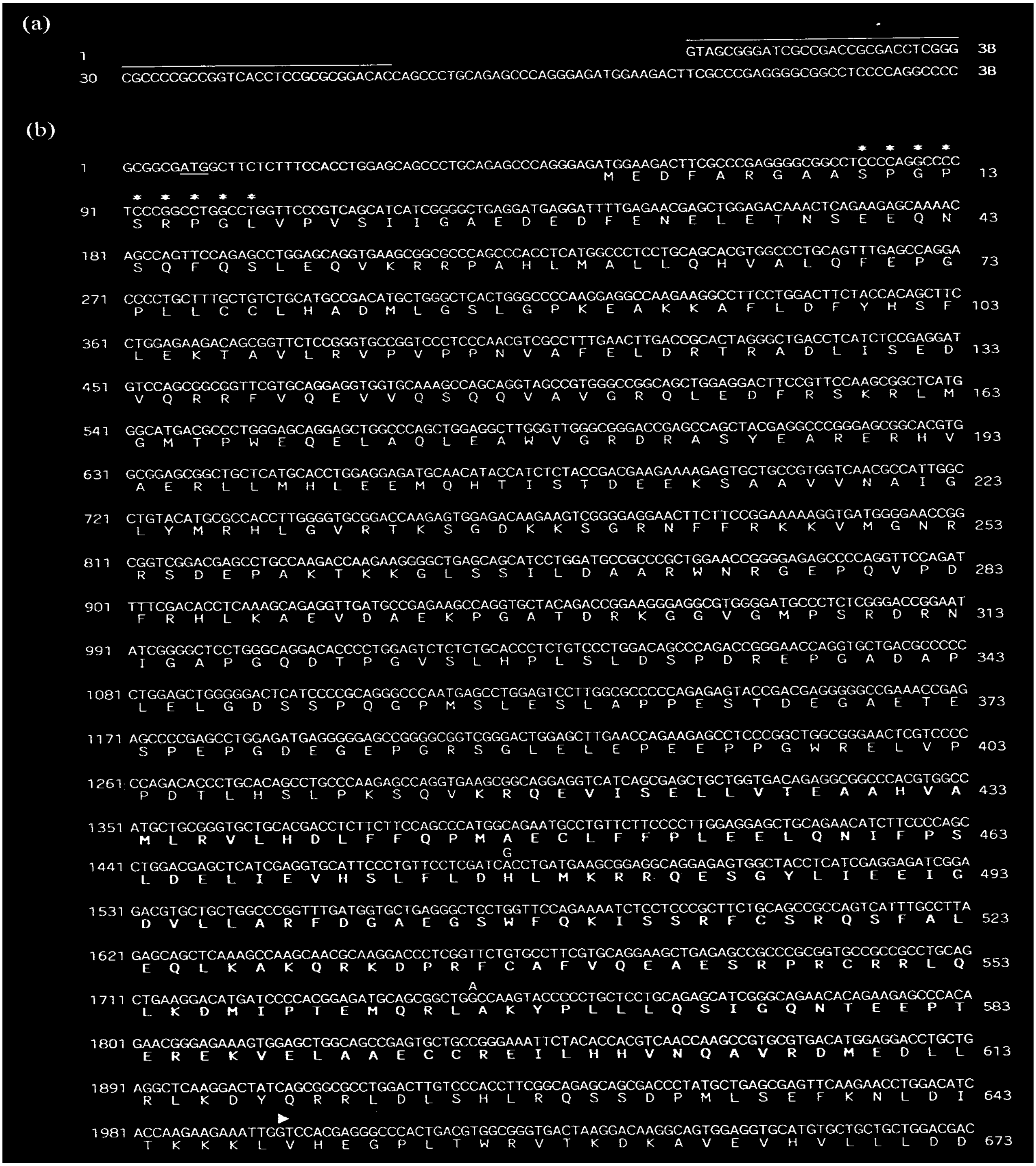

**Fig. 2.** *(a) The alternative 5′ sequence from clone 3B and (b) the nucleotide and predicted amino acid sequence of the lip cDNA clone 11A. 5′ RACE analysis has demonstrated the presence of an additional 18-bp 5′ sequence, GAGGCTTCGGTTCCGGTG, which is missing from clone 11A. The amino acid sequence commences at the second Kozak methionine at nucleotide position 52. The first methionine at position 7 (absent from clone 3B) is underlined, the divergent sequence in clone 3B is overlined. The amino acid substitutions within the DBL homology (DH) domain, introduced by the mismatches between clone 11A and the transfectant clones, are shown above the nucleotide sequence, A → G at nucleotide 1481, G → A at nucleotide 1747. The DH domain is shown in bold. The PH domain is flanked by arrowheads, potential SH3 domain-binding sites are marked by asterisks above the sequence. The positions at which 11A sequence and the sequence in clones T1, T3 diverge are marked with arrows. The stop codon TGA is marked with an asterisk below the sequence. Nucleotide sequence is numbered on the left and amino acid sequence on the right. (c) Nucleotide and predicted amino acid sequence of the 3′ end of transfectant cDNA clone T1. The nucleotide and predicted amino acid sequences are numbered with respect to clone 11A, and sequence common to both clones 11A and T1 is underlined. An arrowhead marks the site of the rearrangement which replaces 188 amino acids from the predicted lip protein with 15 novel residues. The in-frame stop codon is marked with an asterisk. (d) Nucleotide and predicted amino acid sequence of the 3′ end of transfectant clone T3. Nucleotide and amino acid sequences are numbered with respect to clone 11A, and sequences common to both clone 11A and T3 are marked with arrowheads and underlined. The in-frame stop codon is marked with an asterisk. The rearrangement in clone T3 replaces 217 amino acids at the 3′ end of clone 11A with a single novel residue.*

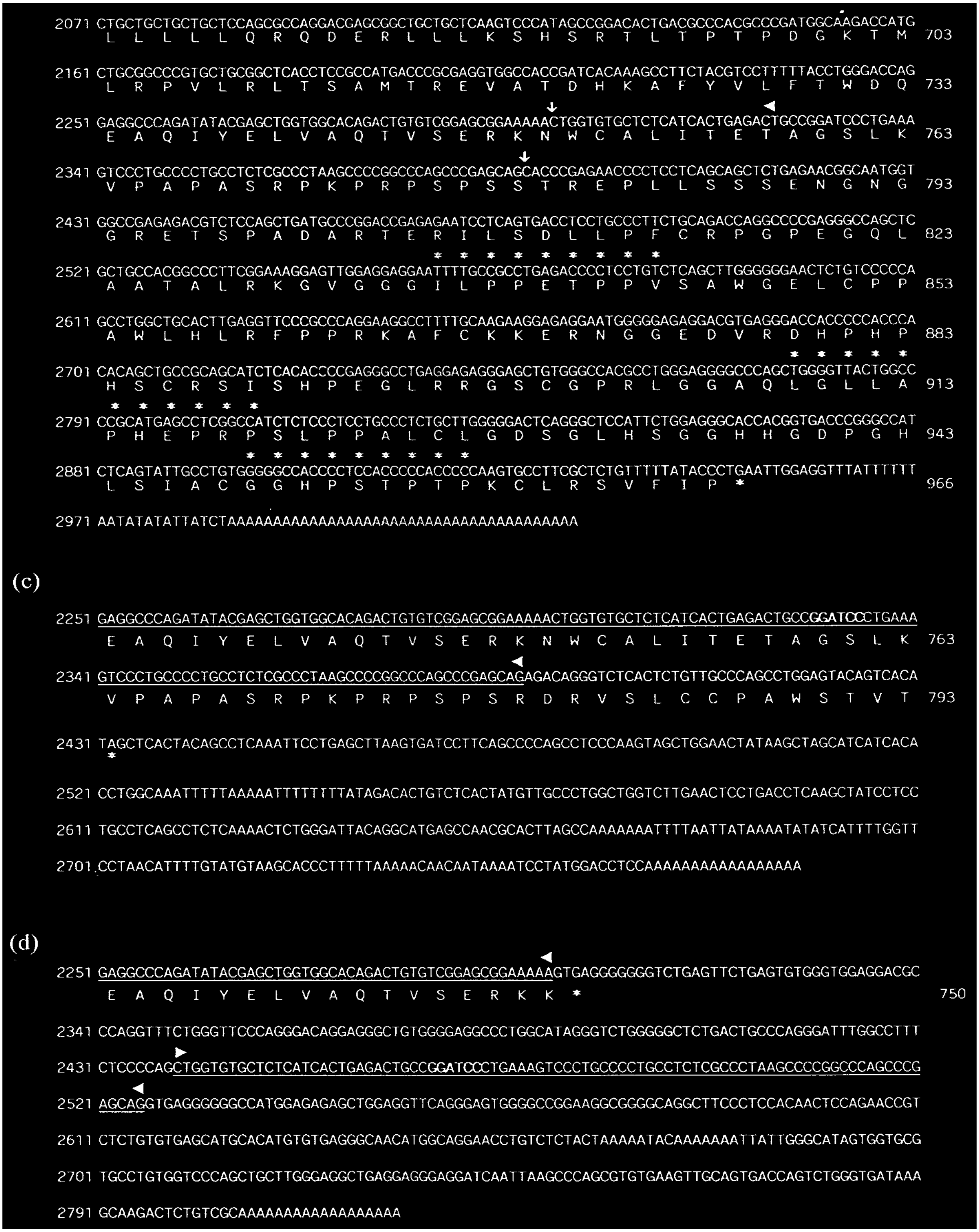

**Fig. 2.** *Continued.*

terminal region. AASPGPSRPGL, which closely resembles the SH3 domain binding sequences seen in the human dynamin protein, a microtubule binding protein which has GTPase activity and is thought to be involved in vesicle trafficking.[27] A Prosite database motif search reveals 13 consensus sequences for protein kinase C phosphoryation, 13 consensus sequences for casein kinase phosphorylation, a single consensus sequence for tyrosine phosphorylation at residue 487 and a single potential site for mitogen-activated protein kinase (MAPK) phosphorylation, P–X–S/T–P, at residue 954 (Fig. 2(b)).

### LIP *shares identity with p115-RHOGEF, an exchange factor for RHOA*

p115-RHOGEF was identified using RHOA as an affinity-purified ligand, and subsequently cloned

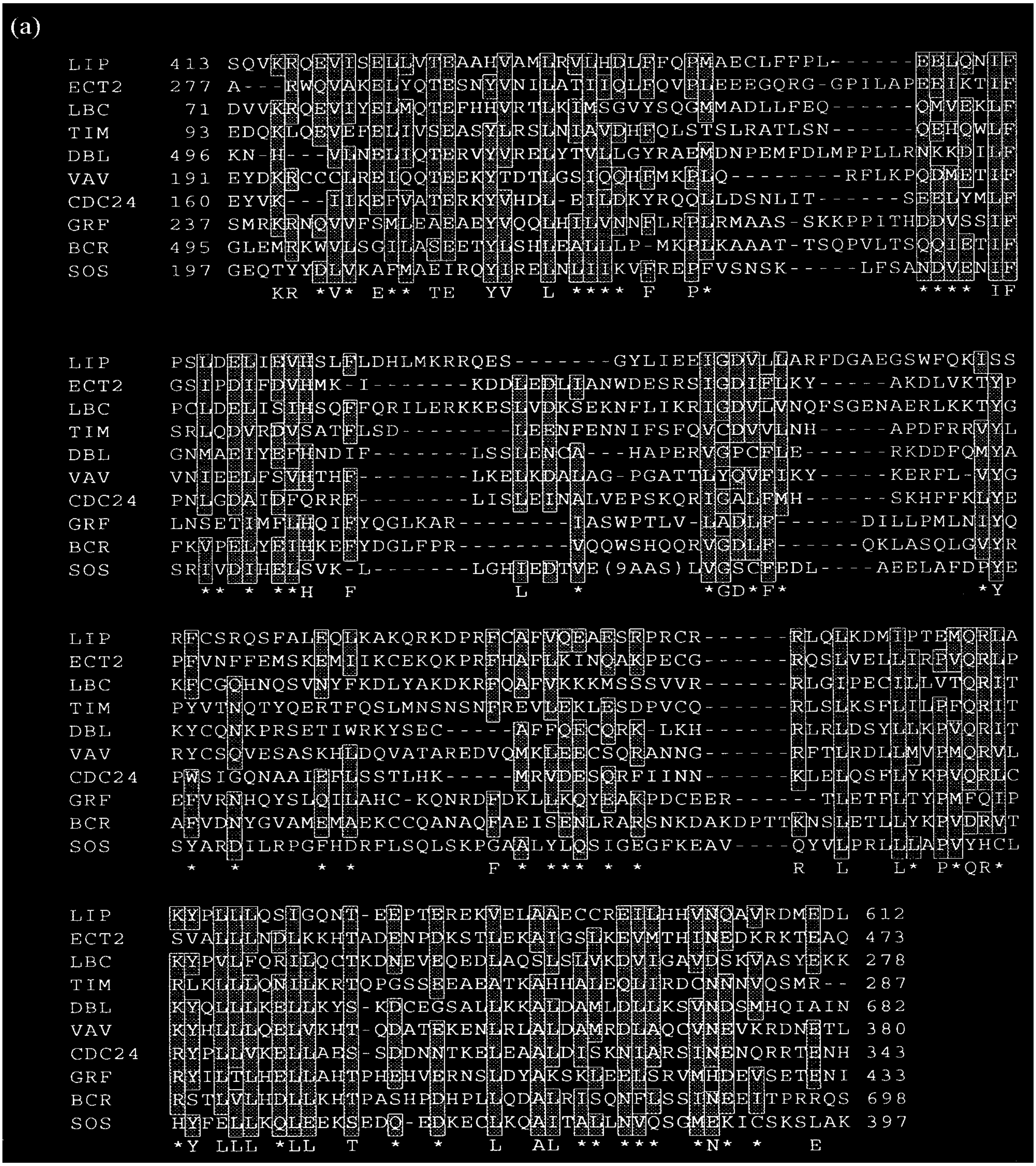

**Fig. 3.** *(a) Computer-generated alignment of the DH domain in LIP, ECT-2, LBC, TIM, DBL, VAV, CDC24, p140RASGRF, BCR and murine SOS using the Clustal V programme. The consensus sequence is indicated below. A capital letter indicates six or more of the proteins possess an identical amino acid at this position, and an asterisk that the amino acid is conserved in six or more of the proteins. Amino acids are numbered on the left. Sequences were obtained from the GENBANK database via Northwick Park Hospital, Middlesex, UK. (b) Alignment of pleckstrin N, pleckstrin C with LIP, VAV, DBL, CDC24, ECT-2, p140RASGRF, BCR and SOS, over the PH domain. The proteins were aligned as previously described.*[30] *The conserved amino acid residues which anchor the five previously described subdomains*[24] *are shown in capitals below the alignment, with additional conserved residues marked with an asterisk.*

from human fetal brain cDNA libraries.[14]. Comparison of the cDNA sequences obtained for *lip* and *p*115-*Rho*GEF shows alternative 5′ untranslated sequences, sequence variations resulting in six isolated amino acid substitutions in the predicted protein sequence and an alternative 3′ end.

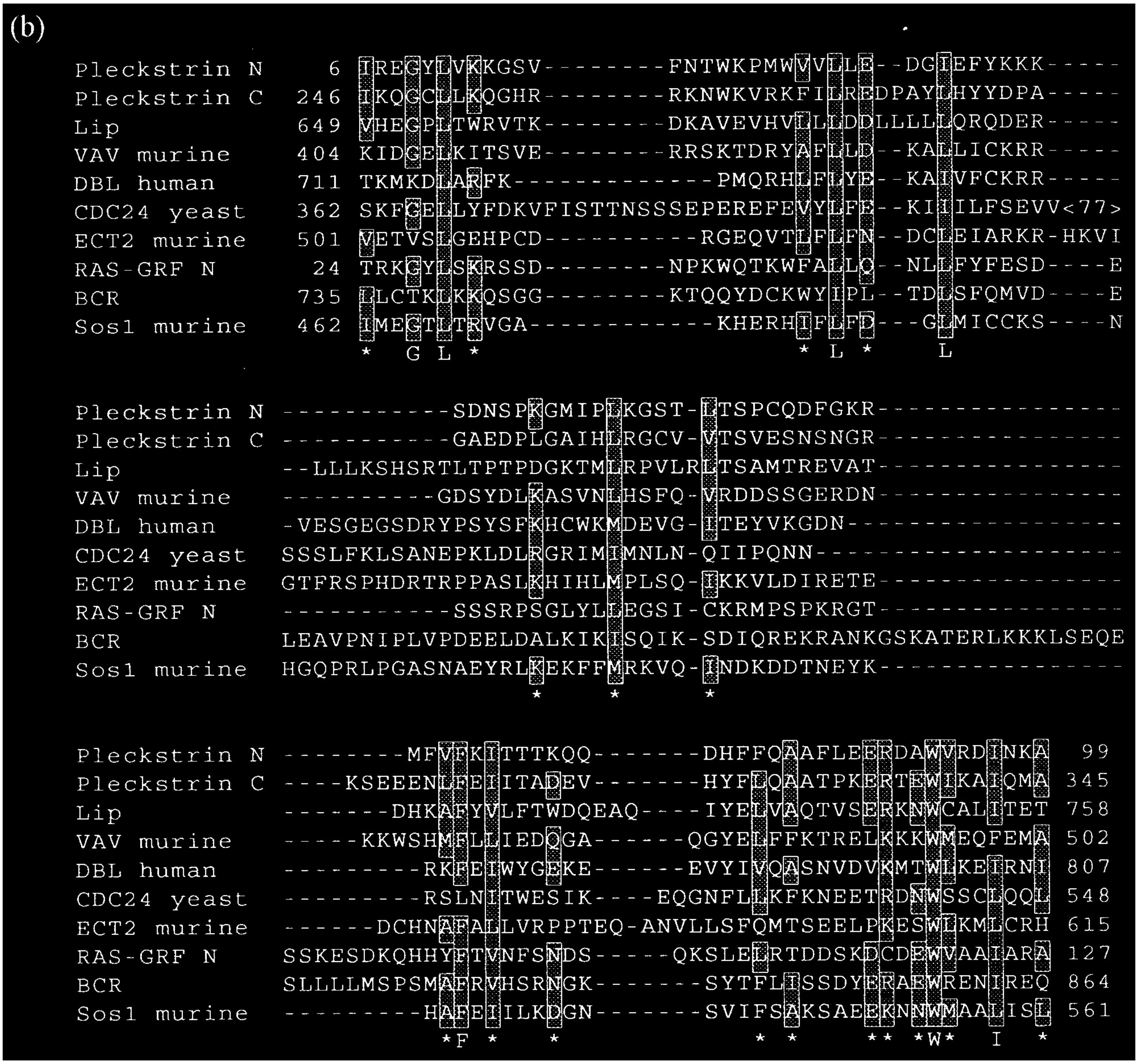

Fig. 3. *Continued.*

The 5′ sequence identified for p115-*Rho*EF is a shortened version of that identified in clone 3B derived from an HT1080 cDNA library, and clone 3B extends this sequence by 26 bp. The alternative 5′ sequence identified in clone 11A and with 5′ RACE analysis using RNA from a human breast cell line contains an in-frame upstream methionine and may represent an alternative start site for translation initiation (Fig. 2b)

With the exception of the six amino acid substitutions, D→E (codon 257), P→A (codon 259), R→A (codon 433), R→H (codon 477), T→A (codon 566) and R→S (codon 776), *lip* and p115-RhoGEF are identical until amino acid 830. At this point the sequence AAA/GGAGTT in *lip* is replaced by AAA/GTGCTG in p115-*Rho*GEF. The sequence in p115-*Rho*GEF is a potential intron splice donor site, and the novel sequences seen in *lip* may have arisen as a result of alternative splicing.

### *Analysis of the lip gene in the primary liposarcoma and in transfectant cell lines*

Hybridization of clone 11A to Southern blots of EcoRI-digested normal genomic DNA gave bands of 15 and 7 kb. These bands were present in tumour DNA from the original liposarcoma, but bands of 17 and 3.7 kb were observed using DNA derived from primary and secondary transfectant cell lines (data not shown). This data indicated that the *lip* gene might be activated by rearrangement, but if this were the case then the rearrangement had taken place during the transfection process and was not present in the primary liposarcoma. To further delineate any role *lip* gene rearrangement might play in sarcoma development, an additional 52 primary tumours were evaluated by Southern analysis of EcoRI-digested tumour DNA. This group included 12 malignant fibrous histiocytomas, 10 leiomyosarcomas, nine liposarcomas, five malignant peripheral nerve sheath tumours, four rhabdomyosarcomas, two synovial sarcormas, one chondrosarcoma, one fibrosarcoma, one post-irradiation spindle cell sarcoma, one dermatofibrosarcoma protuberans, one fibromatosis, two haemangiomas, one lipoma, one neurilemmoma and one clear cell carcinoma. In no case was any rearrangement detected.

*Analysis of the mechanism of lip activation*

To examine the mechanism of activation of *lip*, we prepared an oligo-dT primed cDNA library using RNA from the *lip* transfectant cell line. Screening 200 000 clones using clone 11A as a probe resulted in the identification of 10 partial length cDNA clones (T1 to T10) that were characterized by DNA sequencing. Clone T4 exhibited 88% DNA sequence identity and 98% protein sequence identity to the human *lip* sequence, and probably corresponds to a mouse *lip* clone. Analysis of the remaining clones revealed that they could be divided into three groups, each of which exhibited loss of the 3′ *lip* sequences. In clones T1, 5, 6, 9 and 10, *lip* was replaced 3′ to nucleotide 2387 by a new sequence that in database searches showed regions of homology to human *alu* repeat sequences. This rearrangement replaces 188 amino acids at the carboxy terminus of the predicted *lip* protein with 15 novel amino acids (Figs 1(b) and 2(c)). This result was consistent with Southern analysis data showing that probes prepared from 3′ fragments of the normal *lip* cDNA failed to detect human sequences in *lip* primary and secondary transfectants (results not shown). At position 1747 there was a G→A base change converting an alanine in *lip* to a threonine in the transfectant *lip* clone at codon 566. In addition, T5 which was the longest of all the clones, showed an A→G base change at position 1481 substituting an arginine in *lip* for a histidine in the activated *lip* clone at codon 477. The presence of these alterations, which lie in the DH domain, was confirmed by sequencing a PCR product generated from reverse-transcribed *lip* transfectant RNA, using primers flanking this region.

The second group of cDNAs was represented by a single clone, T3, which also encoded the G→A alteration at codon 1747. In T3, *lip* sequence is interrupted immediately after nucleotide 2300 by 138 bp of novel sequence, followed by an 87-bp sequence corresponding to nucleotides 2301–2387 of *lip* and then by 280 bp of additional novel sequence and a polyA tail. The novel sequences in T3 were unrelated to those in the first group of clones discussed above. In T3, *lip* is truncated by 216 amino acids as a stop codon is introduced immediately following the break-point (Figs 1(b) and 2(d)). This alteration removes an invariant tryptophan from the PH domain discussed earlier. Analysis of the junctions between *lip* sequences and novel sequences in clone T3 suggests that the 138 bp of the novel sequence inserted between nucleotides 2300 and 2301 may be a retained intron (Fig. 1(b)).

The third group of cDNA clones represented by clones T2 and T7 were identical to clone T3 except that their 3′ ends were respectively 133 and 136 bp upstream from the start of the polyA tail in T3.

### Discussion

Here, we describe the cloning and characterization of a gene detected in NIH3T3 transfection experiments using DNA from a human liposarcoma. This gene, *lip*, shares homology with the oncogenes *dbl*, *vav*, *ect-2*, *tim* and *lbc*, and the yeast cell cycle gene *cdc24*, encompassing both the DH and the PH domains. CDC24 functions as an exchange factor for the RHO-like protein CDC42 in budding yeast,[27] DBL is known to act as an exchange factor for CDC42Hs and RHOA,[28] ECT-2 binds RHOC and RAC1[11] and LBC acts as an exchange factor for RHOA, RHOB and RHOC.[29] Moreover, with the exception of six isolated amino acid differences, lip is identical over its N-terminal 830 amino acids (including DH and PH domains) to the recently identified protein p115RhoGEF, which functions as an exchange factor for RHOA.[14] Guanine nucleotide exchange factors bind preferentially to the nucleotide-depleted state of G-proteins, and by stimulating the release of GDP they promote the subsequent binding of GTP, and hence G-protein activation. The DH domain appears to function primarily as an GDP–GTP exchange domain for members of the RHO family. The PH domain[30] appears to be involved in a wide variety of molecular interactions. The C-terminal regions of several PH domains bind to the beta-gamma subunits of heterotrimeric G-proteins,[31,32] and the N-terminal region to phosphoinositol-4,5-bisphosphate,[33] which implies that the PH domain is important for membrane localization. In all the exchange factors which possess both domains, the PH domain is located immediately C-terminal to the DH domain. This suggests that the PH domain is important for the function of the exchange factor domain.

In transfectants, *lip* appears to be activated by C-terminal truncation, a rearrangement which appears to have taken place during the transfection procedure, and was not present in the primary liposarcoma. Moreover, examination of an additional 52 tumours by Southern analysis revealed no evidence in support of a role for *lip* rearrangement in sarcoma development.

Support for C-terminal truncation as the mechanism of *lip* activation comes from evidence that pll5RhoGEF is activated for transformation in the NIH3T3 focus-forming assay by both N- and C-terminal truncation.[14] Moreover, additional members of this protein family have also been shown to be activated by truncation.[8,11,34–36] In general, these activating mutations appear to involve the removal of putative regulatory C-terminal and/or N-terminal domains, but leave the DH and PH domains intact.[37] In the oncogene *lfc*, mutation of the conserved tryptophan residue in the PH domain abolishes transforming activity as does removal of only three amino acid residues from the N-terminal region of its DH domain. Deletions which do not involve the DH or PH domains do not abolish transforming activity. In addition, replacement of the PH domain with an alternative membrane-localizing signal such as an isoprenylation or myristoylation site restores transforming activity.[37] This supports the view that the PH domain is required for DH domain activity, which appears to be promoted by localizing the exchange factor to the plasma membrane.

*lip* is predicted to encode a protein with a number of differences from p115RhoGEF. In addition to differences in six amino acid residues within the N-terminal 830 amino acids. the C-terminal 133 amino acids are unique to *lip*. Within this C-terminal region there are a number of proline-rich regions which conform to the minimal consensus sequence for SH3 domain binding sites.[26] SH3 domain-binding sites have been identified in a number of proteins including, for example, the mammalian RAS exchange factor mSOS.[38] In SOS, these proline-rich sequences mediate binding to the SH3 domains of the adaptor protein GRB2,[39,40] an interaction which recruits SOS to the cell membrane, where it is pivotal in the signal transduction pathway from receptor tyrosine kinases to RAS proteins. LIP and p115RhoGEF may represent alternatively spliced forms of the same gene, and if the proline-rich sequences present in LIP serve a regulatory function, perhaps via a role as SH3 domain-binding sites, then this alternative splicing may represent a mechanism by which LIP/p115RHOGEF function is regulated.

### Acknowledgements

The M426 fibroblast cDNA library was kindly provided by Dr S. Aaronson. This work is supported by grants from the Cancer Research Campaign and the Medical Research Council.

### References

1 Cooper CS, Park M, Blair DG, *et al.* Molecular cloning of a new transforming gene from a chemically-transformed human cell line. *Nature* 1984; 311:29–33.

2 Takahashi M, Ritz J, Cooper GM. Activation of a novel human transforming gene, *ret*, by DNA rearrangement. *Cell* 1985; 42:581–8.

3 Martin–Zanca D, Hughes SH, Barbacid M. A human oncogene formed by the fusion of truncated tropomyosin and tyrosine kinase sequences. *Nature* 1986; 319:743–8.

4 Young D, Waitches G, Birchmeier, *et al.* C. Isolation and characterization of a new cellular oncogene encoding a protein with multiple potential transmembrane domains.*Cell* 1986; 45:711–9.

5 Eva A, Aaronson SA. Isolation of a new human oncogene from a diffuse B-cell lymphoma. *Nature* 1985; 316:273–5.

6 Shimizu K, Nakatsu Y, Sekiguchi, *et al.* Molecular cloning of an activated human oncogene homologous to *v-raf*, from primary stomach cancer. *Proc Natl Acad Sci USA* 1985; 82:5641–5.

7 Taira M, Yoshida T, Miyagawa K, *et al.* cDNA sequence of human transforming gene *hst* and identification of the coding sequence required for transforming activity *Proc Natl Acad Sci USA* 1987; 84:2980–4.

8 Katzav S, Martin–Zanca D, Barbacid M. *vav*, a novel human oncogene derived from a locus ubiquitously expressed in haemopoietic cells. *EMBO* 1989; 8:2283–90.

9 Janssen JWG, Schulz AS, Steenvoorden ACM, *et al.* A novel putative tyrosine kinase receptor with oncogenic potential. *Oncogene* 1991; 6:2113–20.

10 O'Bryan JP, Frye RA, Cogswell PC, *et al. axl*, a transforming gene isolated from primary human myeloid leukaemia cells, encodes a novel receptor tyrosine kinase. *Mol Cell Biol* 1991; 11:5016–31.

11 Miki T, Smith CL, Long JE, *et al.* Oncogene *ect-2* is related to regulators of small GTP-binding proteins. *Nature* 1993; 362:462–5.

12 Toksoz D, Williams DA. Novel human oncogene *lbc* detected by transfection with distinct homology regions to signal transduction products. *Oncogene* 1994; 9:621–8.

13 Gill S, Stratton MR, Patterson H, *et al.* Detection of transforming genes by transfection of DNA from soft-tissue tumours. *Oncogene* 1991; 6:1651–6.

14 Hart MJ, Sharma S, elMasry N, *et al.* Identification of a novel guanine nucleotide exchange factor for the RHO GTPase. *J Biol Chem* 1996; 271:25452–8.

15 Wilkinson M. RNA isolation: a mini-prep method. *Nucleic Acids Res* 1988; 16:10933.

16 Mitchell PJ, Cooper CS. The human *tpr* gene encodes a protein of 2094 amino acids that has extensive coiled-coil regions and an acidic C-terminal domain. *Oncogene* 1992; 7:383–8.

17 Miki T, Matsui T, Heidaran MA, *et al.* An efficient directional cloning system to construct cDNA libraries containing full-length inserts at high frequency. *Gene* 1989; 83:137–46.

18 Chomczynski P, Sacchi N. Single-step method of RNA isolation by guanidinium thiocyanate-phenol-chloroform extraction. *Anal Biochem* 1987; 162:156–9.

19 Sanger F, Nicklen S, Coulson AR. DNA sequencing with chain-terminating inhibitors. *Proc Natl Acad Sci USA* 1977; 74:5463–7.

20 Kozak M. An analysis of 5′-noncoding sequences from 699 vertebrate messenger RNA's. *Nucleic Acids Res* 1987; 15:8125–31.

21 Chan AM-L, McGovern ES, Catalano G, *et al.* Expression cDNA cloning of a novel oncogene with sequence similarity to regulators of small GTP-binding proteins. *Oncogene* 1994; 9:1057–63.

22 Ron D, Zannini M, Lewis M, *et al.* A region of proto-*dbl* essential for its transforming activity shows sequence similarity to a yeast cell cycle gene *cdc24* and the human breakpoint cluster gene *bcr*. *New Biol* 1991; 3:372

23 Shou C, Farnsworth CL, Neel B, *et al*. Molecular cloning of cDNA's encoding a guanine-nucleotide-releasing factor for RAS p21. *Nature* 1992; 358:351–4.

24 Haslam RJ, Koide HB, *et al*. Pleckstrin domain homology. *Nature* 1993; 363:309–10.

25 Tyers M, Rachubinski RA, Stewart MI, *et al*. Molecular cloning and expression of the major protein kinase C substrate of platelets. *Nature* 1988; 333:470–3.

26 Yu H, Chen JK, Feng S, *et al*. Structural basis for the binding of proline-rich peptides to SH3 domains. *Cell* 1994; 76:933–45.

27 Sloat BF, Adams A, Pringle JR. Roles of *cdc24* gene product in cellular morphogenesis during the *Saccharomyces cerevisiae* cell cycle. *J Cell Biol* 1981; 89:395–405.

28 Hart MJ, Eva A, Evans T, *et al*. Catalysis of guanine nucleotide exchange on the CDC42Hs protein by the *dbl* oncogene product. *Nature* 1991; 354:311–4.

29 Zheng Y, Olson MF, Hall A, *et al*. Direct involvement of the small GTP-binding protein RHO in *lbc* oncogene function. *J. Biol Chem* 1995; 270:9031–4.

30 Musacchio A, Gibson T, Rice P, *et al*. The PH domain: a common piece in the structural patchwork of signalling proteins. *M Trends Biol Sci* 1993; 18:343–8.

31 Koch WJ, Inglese J, Stone WC, *et al*. The binding site for the beta-gamma subunits of heterotrimeric G proteins on the beta-adrenergic receptor kinase. *J Biol Chem* 1993; 268:8256–60.

32 Touhara K, Inglese J, Pitcher JA, *et al*. Binding of G-protein beta-gamma subunits to plekstrin homology domains. *J Biol Chem* 1994; 269:10217–20.

33 Harlan JE, Hajduk PJ, Yoon HS, *et al*. Pleckstrin homology domains bind to phosphatidylinositol-4,5-bisphosphate. *Nature* 1994; 371:168–70.

34 Ron D, Tronick SR, Aaronson SA, *et al*. Molecular cloning and characterisation of the human *dbl* proto-oncogene: evidence that its overexpression is sufficient to transform NIH3T3 cells. *EMBO J* 1988; 7:2465–73.

35 Horri Y, Beeler JF, Sakaguchi K, *et al*. A novel oncogene, *ost*, encodes a guanine nucleotide exchange factor that potentially links RHO and RAC signalling pathways. *EMBO J* 1994; 13:4776–86.

36 Habets GGM, Scholtes EHM, Zuydgeest D, *et al*. Identification of an invasion-inducing gene, *tiam-1*, that encodes a protein with homology to GDP-GTP exchangers for RHO-like proteins. *Cell* 1994; 77:537–49.

37 Hart MJ, Sharma S, elMasry N, *et al*. Identification of a novel guanine nucleotide exchange factor for the RHO GTPase. *J Biol Chem* 1996; 271: 25452–8.

38 Bowtell D, Fu P, Simon M, *et al*. Identification of murine homologues of the *Drosophila* son of sevenless gene: potential activators of *ras*. *Proc Natl Acad Sci USA* 1992; 89:6511–5.

39 Egan SE, Giddings BW, Brooks MW, *et al*. Association of SOS RAS exchange protein with GRB2 is implicated in tyrosine kinase signal transduction and transformation. *Nature* 1993; 363:45–51.

40 Rozakis-Adcock M, Femley R, Wade J, *et al*. The SH2 and SH3 domains of mammalian GRB2 couple the EGF receptor to the RAS activator mSOS1. *Nature* 1993; 363:83–5.
